# Underwater endoscopic resection for flat lesions in the stomach often results in immediate bleeding?

**DOI:** 10.1055/a-2601-0106

**Published:** 2025-06-18

**Authors:** Xiaojing Quan, Ying Zheng, Hongbo Wu, Lingzhi Qin, Baicang Zou, Bin Qin

**Affiliations:** 1 117799 Department of Gastroenterology, Xiʼan Jiaotong University Second Affiliated Hospital, Xiʼan, China

Conventional endoscopic mucosal resection (EMR) often presents challenges when treating flat lesions in the stomach. In this study, we successfully performed underwater endoscopic mucosal resection (UEMR) in three cases of flat pre-cancerous lesions located in the gastric body ( Video 1 ).

Underwater endoscopic mucosal resection (UEMR) in three cases of flat pre-cancerous lesions located in the gastric body.Video 1

The first case involved a 71-year-old woman who underwent an outpatient magnified gastroscopy screening for monitoring a flat lesion in the gastric body. During the procedure, a 6 mm flat elevated lesion was identified on the anterior wall of the middle gastric body ( Fig. 1 **a, b** ). Given the economic and time-efficiency advantages over endoscopic submucosal dissection (ESD), UEMR was chosen for this procedure. Despite the use of anesthesia, the patient’s respiratory movements caused significant gastric wall activity. To address this, the lesion was marked with a snare tip ( Fig. 1 **c** ), intraluminal gas was evacuated, and the gastric lumen was immersed in normal saline ( Fig. 1 **d** ). This strategy effectively reduced gastric activity, allowing for easier lesion grasping with the snare. The lesion was successfully resected without perforation, though significant arterial bleeding occurred ( Fig. 1 **e** ), which was promptly managed with electrocautery ( Fig. 1 **f, g** ).

**Fig. 1:**
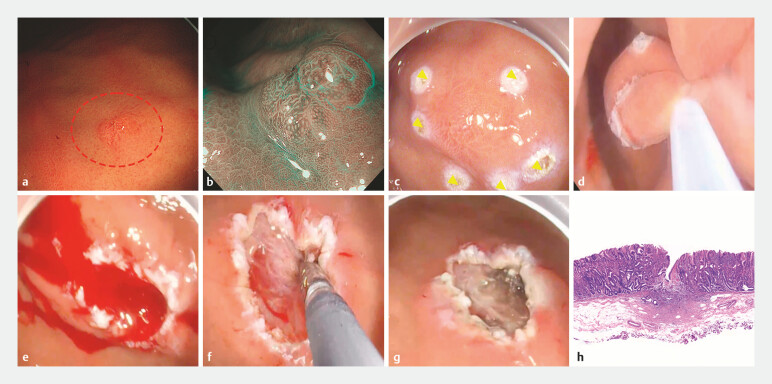
Images of the first case resected via UEMR. **a** Flat elevated lesion on the middle gastric body. **b** Observation of the lesion under chromoendoscopy. **c** Marking of the lesion with a snare tip. **d** Resection of the lesion. **e** Significant arterial bleeding following resection. **f** Immediate control of bleeding using electrocautery. **g** Postoperative wound following successful hemostasis. **h** Postoperative pathology confirming complete resection of the lesion. Abbreviation: UEMR, underwater endoscopic mucosal resection.

The other two cases involved flat lesions located on the greater curvature of the gastric body, each approximately 5 mm in size. Both lesions were successfully resected using UEMR; however, immediate postoperative bleeding occurred in both cases, which was effectively controlled using electrocautery. Pathological examination confirmed adenomas with negative surgical margins in all three cases ( Fig. 1 **h** ).

In summary, UEMR demonstrates an efficient and rapid alternative to ESD for the removal of flat gastric lesions. However, the occurrence of immediate postoperative bleeding highlights the necessity for careful monitoring and prompt intervention.

Endoscopy_UCTN_Code_CPL_1AH_2AZ_3AC

## Notes

Endoscopy E-Videos https://eref.thieme.de/e-videos
*E-Videos* is an open access online section of the journal *Endoscopy* , reporting on interesting cases and new techniques in gastroenterological endoscopy. All papers include a high-quality video and are published with a Creative Commons CC-BY license. Endoscopy E-Videos qualify for HINARI discounts and waivers and eligibility is automatically checked during the submission process. We grant 100% waivers to articles whose corresponding authors are based in Group A countries and 50% waivers to those who are based in Group B countries as classified by Research4Life (see: https://www.research4life.org/access/eligibility/ ). This section has its own submission website at https://mc.manuscriptcentral.com/e-videos .

